# CoQ_10_Phytosomes Improve Cellular Ubiquinone Uptake in Skeletal Muscle Cells: An Ex Vivo Study Using CoQ_10_-Enriched Low-Density Lipoproteins Obtained in a Randomized Crossover Study

**DOI:** 10.3390/antiox12040964

**Published:** 2023-04-20

**Authors:** Fabio Marcheggiani, Patrick Orlando, Sonia Silvestri, Ilenia Cirilli, Antonella Riva, Giovanna Petrangolini, Francesca Orsini, Luca Tiano

**Affiliations:** 1 Department of Life and Environmental Sciences, Polytechnic University of Marche, Via Brecce Bianche, 60131 Ancona, Italyi.cirilli@staff.univpm.it (I.C.); 2 Indena SpA, Viale Ortles, 20139 Milan, Italy; antonella.riva@indena.com (A.R.); giovanna.petrangolini@indena.com (G.P.);

**Keywords:** CoQ_10_phytosome, skeletal muscle, CoQ bioavailability, dermal fibroblasts, CoQ_10_ plasma

## Abstract

Coenzyme Q_10_ (CoQ_10_) bioavailability in vivo is limited due to its lipophilic nature. Moreover, a large body of evidence in the literature shows that muscle CoQ_10_ uptake is limited. In order to address cell specific differences in CoQ uptake, we compared cellular CoQ_10_ content in cultured human dermal fibroblasts and murine skeletal muscle cells that were incubated with lipoproteins from healthy volunteers and enriched with different formulations of CoQ_10_ following oral supplementation. Using a crossover design, eight volunteers were randomized to supplement 100 mg/daily CoQ_10_ for two weeks, delivered both in phytosome form (UBQ) as a lecithin formulation and in CoQ_10_ crystalline form. After supplementation, plasma was collected for CoQ_10_ determination. In the same samples, low density lipoproteins (LDL) were extracted and normalized for CoQ_10_ content, and 0.5 µg/mL in the medium were incubated with the two cell lines for 24 h. The results show that while both formulations were substantially equivalent in terms of plasma bioavailability in vivo, UBQ-enriched lipoproteins showed a higher bioavailability compared with crystalline CoQ_10_-enriched ones both in human dermal fibroblasts (+103%) and in murine skeletal myoblasts (+48%). Our data suggest that phytosome carriers might provide a specific advantage in delivering CoQ_10_ to skin and muscle tissues.

## 1. Introduction

Ubiquinone or Coenzyme Q_10_ (CoQ_10_) is a ubiquitous, endogenous lipophilic cofactor that is well known for its bioenergetic and antioxidant functions within cells. In particular, CoQ_10_ as a component of the mitochondrial electron transport chainacts as an electron shuttle between complex I, II, and complex III [1,2,3,4]. Within the mitochondria, CoQ_10_ has been shown to modulate the permeability transition pore and activity of uncoupling protein [5,6,7]. Moreover, CoQ_10_ in its reduced form ubiquinolis endowed with antioxidant properties in all biological membranes and acts synergistically with vitamin E and vitamin C to protect against lipid peroxidation [2].

Mitochondria-rich skeletal and cardiac muscle tissues have the highest content in CoQ_10_ compared with other tissues, and its levels are shown to be directly related to muscle functionality. Moreover, CoQ_10_ content is decreased in these tissues in senescence as well as under pathological conditions which has important prognostic implications [8]. For these reasons, numerous CoQ_10_ interventions have been designed to contrast cardiovascular pathologies in relation to both its bioenergetic function as well as antioxidant and anti-inflammatory activities [9].

In the skeletal muscle, several CoQ interventions investigated its role as a dietary supplement in sport nutrition as an antioxidant to contrast physical exercise-induced oxidative stress or as a bioenergetic support [10,11].

In fact, although a healthy organism produces sufficient amounts of CoQ_10_ for its normal function, secondary CoQ deficits are not uncommon and are related to increased consumption duringintense physical exercise [12], pathologically impaired redox status, and lowered biosynthesis that is associated with senescence [13,14,15]. Dietary intake accounts for approximately −5 mg/day [16]. CoQ-rich foods include meat, fish, and nuts, and the highest concentration of CoQ is found in heart tissue [16]; however, that is not frequently consumed in the human diet.

Moreover, CoQ in general shows limited gastrointestinal absorption and bioavailability due to its chemical–physical properties [17]. Dietary CoQ_10_ distribution shows tissue specific differences in uptakewith plasma showing the most increases and muscle tissue showing the least [18,19]. This represents a unique feature of CoQ_10_ that behaves differently from similar lipophilic molecules such as tocopherol that seem to be less tissue specific [20].

Accordingly, understanding the molecular mechanisms regulating CoQ uptake in the muscle represents a priority for the realization of innovative formulations that could promote enhanced bioavailability in these refractory tissues. A recent study by Drobnic et al. [21] did show that CoQ_10_ formulated in phytosomes as Ubiqsome^®^ (standardized in the coenzyme Q_10_18–22% by HPLC), led to a significant increase in quinone in skeletal muscle tissue in vivo.

**Figure 1:**
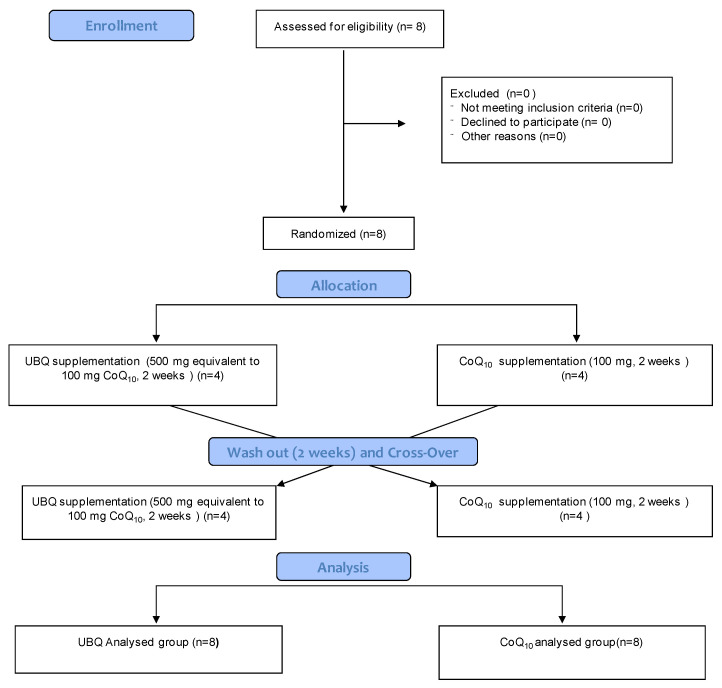
CONSORT flowchart. A total number of 8 patients were enrolled. Volunteers were randomized in 2 groups (CoQ_10_, crystallized CoQ_10_ and UBQ, Ubiqsome) according to a crossover design. For for LDL extraction plasma was subsequently pooled into 2 groups, each one containing plasma from 4 patients.

The present pilot study exploits anex vivodesign to verify the bioavailability of CoQ_10_ phytosome compared with crystalline CoQ_10_ in different cellular models characterized by a different ability to absorb exogenous CoQ_10_—namely, dermal fibroblasts and skeletal myoblasts. In order to mimic thein vitroinvestigation within vivoprocessesas much as possible, CoQ_10_ was given in the form of enriched lipoproteins that were normalized in their CoQ_10_ content following HPLC determination and appropriately diluted in cell culture medium to 0.5 µg/mL, as shown in Figure 1.

## 2. Materials and Methods

### 2.1. Experimental Design

This human study was conducted between September and November 2022 using atwo arms randomized crossover design with a wash-out phase that enrolled 8 subjects following the layout reported in Table 1. Specifically, the enrolled subjects were 4 males (age 32 ± 2) and 4 females (age 29 ± 1). The inclusion criteria included healthy subjects aged <40 years with a BMI between 18.5 and 24.9 kg/m^2^.

At the beginning of the study, the participants were randomized into two groups using the free Research Randomizer tool (www.randomizer.org accessed on 1 September 2022). One group received 500 mg/day of phytosome Coenzyme Q_10_ in capsules (UBQ) for two weeks, which is equivalent to 100 mg of CoQ_10_ (INDENA, Italy), and the second group received 100 mg/day of crystallized CoQ_10_ in capsules (CoQ)for two weeks. Both crystalline and Ubiqsome were provided by INDENA. Containers that were labelled as A or B were sequentially numbered and matched with the randomization list and were provided to blinded operators who allocated the containers to volunteers. The generation of randomization codes and the labelling of containers was conducted by independent operators who were not directly involved in the experimental procedures. Volunteers and researchers were blinded to the allocation sequence, and plasma and LDL extracts were classified based on generic labelling (A or B) in order to allow LDL pooling for exvivo study. Volunteers were instructed to take supplements with a meal. None of the participants took any medication or dietary supplements within 1 month before the beginning of the study. Following the two weeks of supplementation, the volunteers had two weeks of a washout phase. Subsequently, the groups were inverted so that all the subjects involved in the study took both formulations at subsequent times. The primary endpoint of thein vivo clinical study was the evaluation of Q_10_ plasma bioavailability. The isolated lipoproteins were subsequently used for exvivo studies in the cell culture models described.

### 2.2. Blood Samples and Extraction of Enriched LDL from Plasma

Blood (20 mL) was collected under fasting conditions in lithiumheparin vacutainers from each subject at the base line and at the end of each treatment phase at the Department of Life and Environmental Sciences (DISVA, UNIVPM) by qualified operators. Plasma (approximately 12 mL from each subject) was obtained by centrifugation at 1600× *g* for 5 min at 4 °C within 20 min after blood withdrawal. 250 µL of plasma was immediately stored at −80 °C to evaluate the CoQ_10_ amount and oxidative status forin vivobioavailability determination. The remaining fresh plasma was pooled among the volunteers taking the same formulation within the same arm of the study.

Subsequently, low-density lipoproteins were extracted from the pooled plasma using a heparin trisodium citrate solution, as previously reported by Weiland et al. [22], and the insoluble LDLs’ pellets were resuspended in cold PBS (0.1 M sodium phosphate buffer, pH7.4, containing 0.9% NaCl).In order to purify and concentrate the LDL, the solution was centrifuged and filtered at 2900 g for 20 min at 10 °C in a tube equipped with a filter membrane with a 10,000 kDa cutoff (centrifugal filter devices Amicon Ultra 4 mL, Millipore, Burlington, MA, USA). The enriched LDL samples were divided into 2 mL aliquots and stored at −80 °C in order to limit freeze–thawing procedures that are known to affect LDL oxidation.

**Figure 2:**
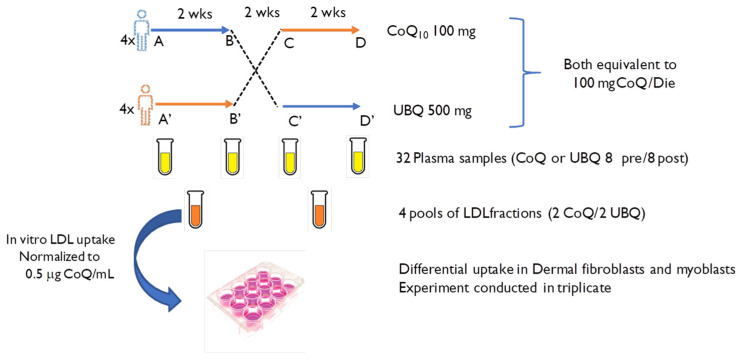
Layout of the study design. A and A’: pre-supplementation; B and B’: post-supplementation; C and C’: pre-supplementation after wash-out; D and D’: post-supplementation post wash-out. After collecting the blood samples, a total of 32 plasma samples were obtained and divided into 16 plasma samples (pre- and post-supplementation) each for crystalline CoQ_10_ (CoQ) and Ubiqsome (UBQ). From these plasma samples, 4 pools of enriched LDLs were extracted and divided into 2 each for CoQ and UBQ.

In vitrostudies were conducted using 4 pools of LDL fraction, including 2 for each formulation, as reported in Figure 2. In particular, enriched LDLs were derived from 8 plasma aliquots B, D (CoQ_10_ crystallized post-treatment) and 8 plasma aliquots B’, D’ (Ubiqsome post-treatment).

### 2.3. Cell Culture and LDL Treatment

Human dermal fibroblasts (HDF) were purchased from the Istituto Zooprofilattico Sperimentale (Brescia, Italy) as a pooled sample from female subjects (40 years).HDFs were cultured in MEM with Earl salts (Carlo Erba, Italy) that were supplemented with 10% fetalbovine serum (South American Origin, Euroclone, Pero, Italy), 1% antimycin (10,000 U/mL) and streptomycin (10 mg/mL), stable glutamine (200 mM), and amphotericin B (250 µg/mL, Euroclone, Pero, Italy) and maintained in a 5% CO_2_ atmosphere at 37 °C. The complete medium was replaced every two days.

Mouse immortalised skeletal muscle myoblasts (C2C12), kindly provided by Prof. Michele Guescini, University of Urbino (Italy), were cultured in Dulbecco’s Modified Eagle Medium (DMEM) which was supplemented with 10% heat-inactivated fetal bovine serum (FBS, South American Origin, Euroclone, Pero, Italy), 1% glutamine (200 mM), 1% penicillin (10,000 U/mL), and 1% streptomycin (10 mg/mL) and maintained in a 5% CO_2_ atmosphere at 37 °C.

The LDL treatment was performed for both cell types at the sub-confluence state. Pooled LDLs with different formulations (UBQ and CoQ) were added to the complete medium to reach equal ubiquinol (QH) concentration (0.5 µg/mL). To avoid external contaminations, the solutions of media that were supplemented with pooled LDL were filtered through a 0.2 µm filter using a syringe and subsequently injected into the HPLC system to verify whether the ubiquinol concentrations were affected by filtration. Cells treated with only the complete medium were used as negative controls. All experiments were conducted independently three times.

### 2.4. Coenzyme Q_10_Level and Its Oxidative Status Determination in Plasma and Cell Samples

Plasma and cellular CoQ_10_ content and their oxidative statuses were analyzed by electrochemical detection using high performance liquid chromatography (Nanospace HPLC-ECD, Shiseido, Tokyo, Japan) associated with a post-chromatographic reducing column (Shiseido CQR, Tokyo, Japan) that simultaneously measured both the oxidized and reduced forms of CoQ, as previously described [12]. In particular, to quantify the total CoQ (CoQ_9_ + CoQ_10_) in the mouse myoblasts, the HPLC method, described by Andreani et al. [23], was used to discriminate the four peaks relative to the reduced and oxidized forms of CoQ_9_ and CoQ_10_.

CoQ_10_ plasma content was expressed as total plasma CoQ_10_ levels (µg/mL) or plasma nmol CoQ_10_/mmol cholesterol. The CoQ oxidative status was expressed as the percentage of oxidized CoQ with respect to the total CoQ.

Cellular CoQ levels were normalized by protein content that was assessed using the BCA protein assay kit (Thermofisher, Waltham, MA, USA), and content was expressed as CoQ_10_ ng/mg protein or total CoQ ng/mg protein.

### 2.5. Sample Size and Statistical Analysis

Sample size determination was based on mean values observed in similar studies that were conducted in our laboratory by evaluating the plasma bioavailability of different CoQ_10_ formulations.

In particular, the expected mean difference was used as a reference value. Considering a mean plasma change of 0.5 ± 0.34 µg/mL in the treated groups, 8 subjects would be required to detect a difference with 80% power and a 5% two-sided type I error rate.

Data from primary outcomes were expressed as means (SDs) and followed a normal distribution; therefore, unpaired *t*-tests with Welch’s correction were used. Two-way ANOVA was performed on the exvivo study in cell culture models using Tukey’s multiple comparisons test to compare control (Ctrl) with CoQ_10_ cells. Statistical significance was defined as a two-sided *p* value < 0.05.

## 3. Results

### 3.1. Both Crystalline CoQ_10_ and Ubiqsome Supplementation Produced a Significant Increase in CoQ_10_Plasma Levelsand Improved Its Oxidative Status

**Figure 3:**
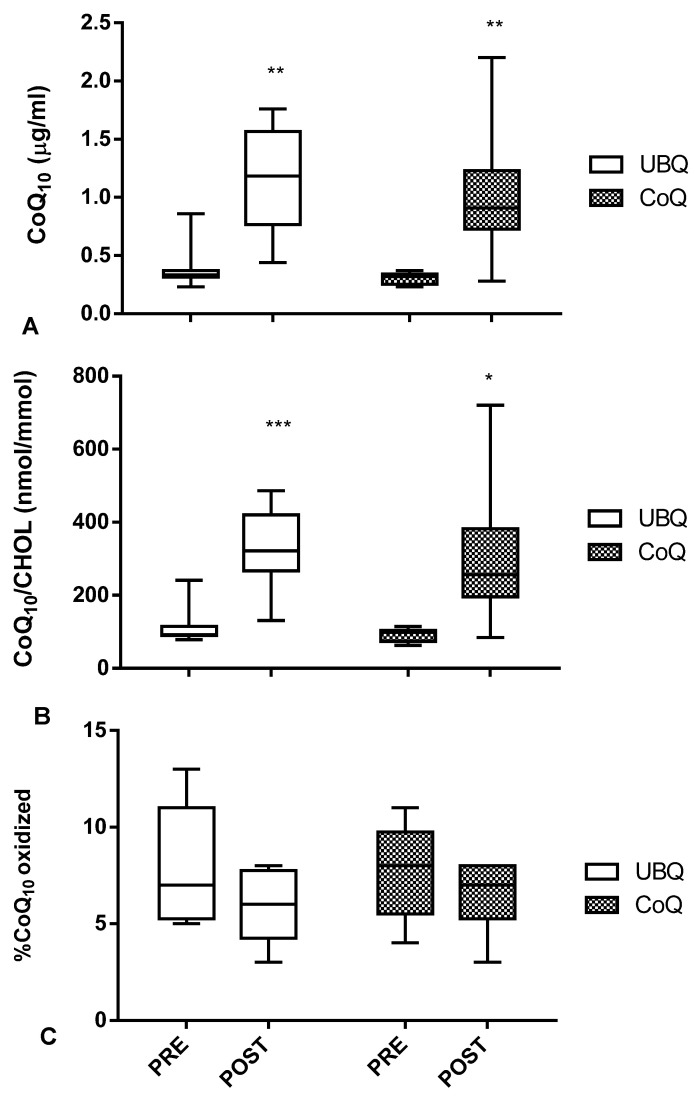
Plasma CoQ_10_ levels. (**A**) CoQ_10_ normalized to cholesterol (CHOL) levels (**B**) and percent of oxidized CoQ_10_ (**C**) in human healthy volunteers supplemented for 2 weeks with UBQ and crystalline CoQ_10_ (CoQ) at the same dose (i.e., 100 mg/day of CoQ_10_). Data are expressed as boxes and bar plots (mean value) (*n* = 8). Statistical significance was calculated using unpaired *t*-tests with Welch’s correction compared with pre-supplementation (PRE) (* *p* < 0.05, ** *p* < 0.01, *** *p* < 0.001).

The consort 2010 flow chart describing the different steps of the invivo study is reported in Figure 1. All volunteers who participated in the study successfully completed the trial and no withdrawal or any side effects were reported. All data reported in theinvivostudy refers to the mean of eight values at each time point taking into consideration the crossover design. Two weeks of supplementation with UBQ resulted in a significant absolute plasma CoQ_10_ increase from 0.4 ± 0.2 µg/mL to 1.2 ± 0.5 µg/mL (*p* < 0.01) (Figure 3A). Crystalline CoQ_10_ supplementation also produced a significant increase in plasma levels (from 0.3 ± 0.1 µg/mL to 1.0 ± 0.6 µg/mL (*p* < 0.01). The total amount of plasma CoQ_10_ levels in each volunteer ranged from 0.2 to 0.9 µg/mL for basal conditions, while plasma CoQ_10_ levels ranged from 0.4 to 2.2 µg/mL following supplementation.

Since CoQ_10_ is transported in plasma by lipoproteins, mainly LDL, variation in cholesterolemia could affect absolute CoQ_10_ plasma determination. Nonetheless, a similar response was also observed following total cholesterol normalization in plasma CoQ_10_ normalized to cholesterol, with variations for UBQ and crystalline CoQ_10_changing from 114 ± 53 nmol CoQ_10_/mmol CHOL to 320 ± 111 nmol CoQ_10_/mmol CHOL (+181%, *p* < 0.001) and from 93 ± 18 nmol CoQ_10_/mmol CHOL to 312± 192 nmol CoQ_10_/mmol CHOL (+235%, *p* < 0.05), respectively (Figure 3B).

Dietary supplementation also resulted in a slight improvement in plasma CoQ_10_ oxidative status. The baseline percentage of oxidized CoQ_10_ in volunteers was 8%. The percentage of oxidized CoQ_10_ decreased following UBQ (6%) and crystalline CoQ_10_ (6.5%) supplementation, while no significant differences were detected between the two types of intervention (−0.5%; *p* = 0.51) (Figure 3C).

Indeed, the large majority of exogenous CoQ_10_ upon transfer through the gastrointestinal barrier is converted to ubiquinol and incorporated in lipoproteins. This biochemically mediated invivo transformation of ubiquinone to ubiquinol, together with the complexity of the lipoprotein environment, represents a critical advantage of the exvivo experimental design used in the present study.

### 3.2. CoQ_10_-Enriched LDL from Ubiqsome Supplemented Subjects Are More Efficient Vectors of CoQ_10_ to Cultured Cells

**Figure 4:**
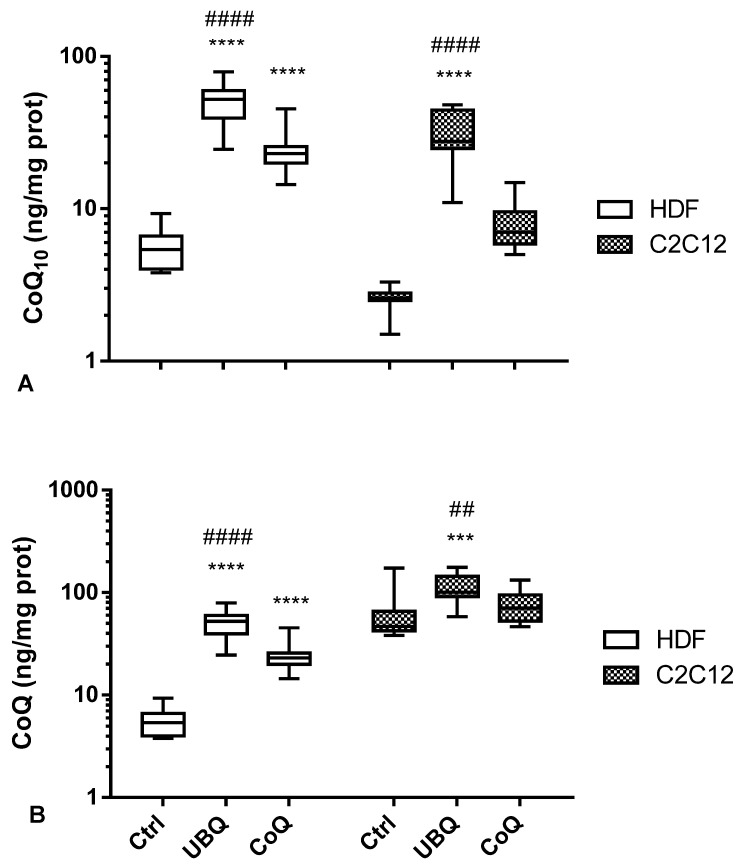
Cellular CoQ_10_ (**A**) and total CoQ levels (**B**) in human dermal fibroblasts (white clear box plots) and murine skeletal myoblasts (small dot box plots) treated with p medium (controls, Ctrl), enriched LDL derived from the plasma of volunteers supplemented with Ubiqsome (UBQ) or crystalline CoQ_10_ (CoQ) at the same concentration of CoQ_10_ 0.5 µg/mL for 24 h. Data are expressed as boxes and bar plots (mean value) (*n* = 3). Statistical significance was calculated using two-way Anova with Tukey’s multiple comparisons tests compared with the control cells (Ctrl) (*** *p* < 0.001, **** *p* < 0.0001) or crystalline CoQ_10_ (CoQ) (## *p* < 0.01, #### *p* < 0.0001).

The incorporation of exogenous CoQ_10_ was quantified by HPLC following 24 h incubation both in human dermal fibroblasts and mouse skeletal myoblasts (Figure 4A,B). The results demonstrate that LDL from UBQ-supplemented volunteers were able to better deliver CoQ_10_ in both cellular models.

In particular, in human dermal fibroblasts exposed to LDL from UBQ-supplemented subjects, cellular CoQ_10_ increased by 9-fold compared withthe basal level recorded in untreated cells as it went from 5.6 ± 1.6 ng CoQ/mg protein to 51.4 ± 15.6 ng CoQ/mg prot (*p* < 0.0001) (Figure 4A). Significantly lower increases (4.5-fold; *p* < 0.0001) were observed in the same dermal fibroblasts incubated for the same time and at the same concentration of CoQ in the presence of LDL isolated from crystalline CoQ_10_-supplemented subjects. While the total increase was also substantially halved in this case, a highly significant increase from the baseline values was recorded, with cellular CoQ content ranging in this case from 5.6 ± 1.6 ng CoQ/mg protein to 25.3 ± 9.8 ng CoQ/mg protein (*p* < 0.0001) (Figure 4A).

An even more divergent behavior in CoQ_10_ delivering efficacy between LDL from UBQ- and CoQ-supplemented subjects was observed in murine skeletal myoblasts. In these cells, 24 hrs incubation with cell culture medium containing 0.5 µg CoQ_10_/mL from UBQ-enriched LDL produced a 12-fold increase in cellular CoQ_10_ content increasing from 2.6 ± 0.5 ng CoQ_10_/mg protein to 30.2 ± 12.6 ng CoQ_10_/mg protein (*p* < 0.0001) (Figure 4A). On the contrary, myoblasts incubated under the same experimental conditions using LDL from CoQ_10_-supplemented volunteers increased the cellular CoQ_10_ amount only 3-fold (from 2.6 ± 0.5 ng CoQ_10_/mg protein to 7.9 ± 2.8 ng CoQ_10_/mg protein; *p* = 0.34) (Figure 4A). In summary, in murine skeletal myoblasts, UBQ formulation was 4-fold more bioavailable in comparison with crystalline CoQ_10_ (*p* < 0.0001) (Figure 4A).

The murine cells data described in this study refer only to the CoQ_10_ fraction which is a minor component of the total Coenzyme Q pool of these cells, as shown in Table 1. Indeed, it is important to note that two cellular models, human and murine, are different in terms of their CoQ composition. While human cells contain only CoQ_10_, on the contrary, murine cells contain mainly CoQ_9_ and a far lower content of CoQ_10_. Interestingly, if we consider the total cellular CoQ content in murine cells (CoQ_9_ + CoQ_10_), as expected, this is higher in mitochondria rich skeletal muscle cells (66.1 ± 43.2 ng CoQ/mg protein) compared with dermal fibroblasts (5.6 ± 1.6 ng CoQ/mg prot).

Following incubation with LDL from the UBQ-supplemented subjects, the total CoQ cellular content in the skeletal muscle cells increased to 113.5 ± 37.4 ng CoQ/mg protein (1.7-fold increase, *p* < 0.001) (Figure 3B). However, in the same cells incubated with LDL from crystalline CoQ_10_-supplemented subjects, the total CoQ content increased only to 76.8 ± 30.5 ng CoQ/mg protein (1.2-fold increase, *p* = 0.62). In conclusion, in relation to total CoQ cellular content in skeletal myoblast, UBQ was 1.4-fold more bioavailable in comparison with crystalline CoQ_10_ (*p* < 0.0001) (Figure 4B).

### 3.3. Ubiqsome Enriched LDL Were More Effective in Improving Cellular CoQ_10_Oxidative Status in Murine Myoblasts

**Figure 5:**
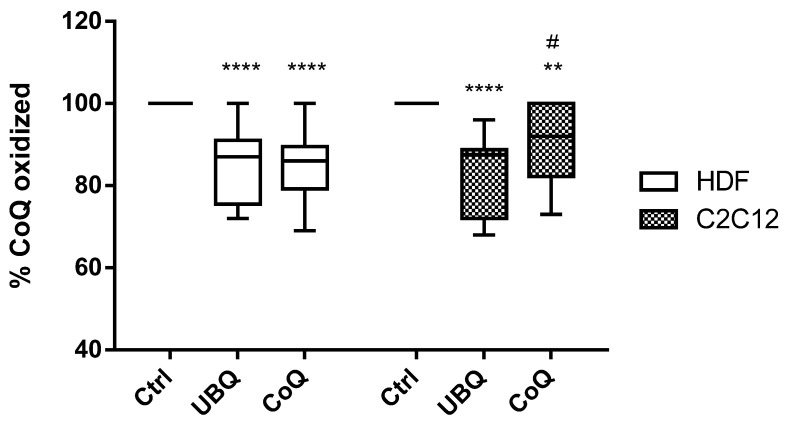
Cellular CoQ_10_ oxidized levels in human dermal fibroblasts (white clear box plots) and mouse skeletal myoblasts (small dot box plots) treated with medium (controls, Ctrl), enriched LDL derived from the plasma of subjects supplemented with Ubiqsome (UBQ) or crystalline CoQ_10_ (CoQ) at the same concentration of CoQ_10_ 0.5 µg/mL for 24 h. Data are expressed as boxes and bar plots (median value) (*n* = 3). Statistical significance was calculated using two-way Anova with Tukey’s multiple comparisons tests compared with the control cells (Ctrl) (** *p* < 0.01, **** *p* < 0.0001) or crystalline CoQ_10_ (CoQ) (# *p* <0.05).

In untreated human dermal fibroblasts, the whole CoQ content in cellular extracts was in the oxidized form (Figure 5). Supplementation with UBQ- or crystalline CoQ_10_-enriched LDL was able to decrease cellular CoQ_10_ oxidation to 85 ± 8% (*p* < 0.0001) (Figure 5), and no significant differences were observed between the two interventions (*p* = 0.99).

When mouse skeletal myoblasts were treated with enriched LDL, the difference between the two formulations was detectable. In fact, in crystalline CoQ_10_-treated cells, the level of oxidation was 91 ± 10 (Figure 5, *p* < 0.01 compared with the baseline), while in the UBQ-exposed myoblasts, the percentage of oxidized CoQ was 83 ± 10% (*p* < 0.0001 compared with the baseline; *p* < 0.014 compared with crystalline CoQ_10_).

## 4. Discussion

Coenzyme Q_10_ is an established nutritional supplement for improving human health in different clinical conditions. Indeed, CoQ_10_ secondary deficiency is not a rare condition and is associated with different pathological conditions characterized by increased oxidative stress [24], such as cardiovascular and metabolic diseases [25,26], drugs interfering with its biosynthesis, such as statins [27,28], aging, and senescence-related organ dysfunction, such as sarcopenia [24,29].

The beneficial effects of CoQ_10_ are well documented, both as a potent antioxidant in the lipid environment [2], as an anti-inflammatory agent [30,31], as well as a promotor of mitochondrial energy metabolism [32,33].

However, a major issue associated with CoQ_10_ use in clinical settings is related to its poor bioavailability and limited cellular uptak; in particular, muscle and cardiac tissues are the most refractory tissues to exogenous CoQ_10_ uptake [23,34], and the efficacy of oral supplementation in these districts is highly debated [23,34,35]. Onlyone study byKamzalovet al. observed a significant increase in muscle CoQ_10_ derived from homogenate and mitochondria in mice that were treated with CoQ_10_ at 148 and 654 mg/kg for 11 weeks [36].

Accordingly, several research efforts have addressed the topic of enhancing CoQ_10_ bioavailability; in particular, in terms of the development of novel delivery formulas, particle size reduction (nanoparticles), the solid dispersion of water insoluble drugs, microemulsion systems, cyclodextrin complexes, and liposomes [37,38,39]. In the present study, we investigated a new form of carrier involving a lecithin-based formulation also known as Phytosome. Phytosomes represent a functional solid dispersion in a phospholipid matrix. Ubiqsome is a phytosome standardized in coenzyme Q_10_18–22% by HPLC which previously showed enhanced plasmatic levels of CoQ_10_ after administration in a single dose [40].

Ubiqsome formulations have already been tested in invitro models using rat cardiac and human epithelial cells in relation to tissue CoQ_10_ bioavailability, cellular redox state, and cellular bioenergetics in comparison with pure CoQ_10_ form. Notably, 100 nM of Ubiqsome for 24 h was able to increase cellular and mitochondrial CoQ_10_ content that is associated with increased antioxidant defences (decreased lipid membrane peroxidation and ferroptosis) and improved mitochondria functionality (increased ATP production, spared respiratory capacity, and mitochondrial membrane potential) in both cell lines. On the contrary, the same dose of pure CoQ_10_ did not show any increase in tissue CoQ_10_ bioavailability, cellular antioxidant capacities, or bioenergetic parameters. Additionally, the authors reported that tissue CoQ_10_ uptake in both cell lines likely involved macropinocytosis mechanisms [41].

Moreover, in a recent study conducted in healthy aged athletes exposed to intense physical exercise, UBQ supplementation (equivalent to 100 mg CoQ_10_ per day for 1 month) was able to increase both plasma and muscular CoQ_10_ content. In particular, the study reported a remarkable and unique 36% increase in muscle CoQ_10_ levels following supplementation with UBQ [21]. In the supplemented subjects, increases in CoQ_10_ content were associated with protection from physical exercise-induced oxidative damage (decline in plasma malonyl dialdehyde levels) and a concomitant increase in plasma total antioxidant capacity. Moreover, the exercise-induced proinflammatory plasmatic markers IL6 and IL10 also significantly decreased.

In order to further investigate the peculiar bioavailability of Ubiqsome in muscle cells in more detail, we developed anex vivostudy design involving, as a first step, the oral supplementation of healthy subjects with either a standard crystalline CoQ_10_ formulation or Ubiqsome using equimolar doses of CoQ_10_ (100 mg/day for 2 weeks). Subsequently, CoQ_10_-enriched LDL deriving from subjects after supplementation with both products were used to treat human dermal fibroblasts known to be able to incorporate efficiently exogenous CoQ_10_ and murine myoblasts that, on the contrary, are more refractory to exogenous CoQ_10_ uptake. The proposed experimental model is able to mimic, in a more rigorous approach, the physiological exposure of tissues to CoQ_10_ that are essentially transported by lipoproteins. This model incorporates all biological transformations that take place at the gastrointestinal layer, such as a reduction in CoQ_10_ and lipoprotein assembly, and systemically, such as the maturation of LDL that constitute the primary carriers of CoQ_10_ in the circulation.

Our data are in agreement with the results obtained in an in vivo study conducted by Drobnic et al. [21] and may provide a simple approach to compare the cellular bioavailability of CoQ_10_ from LDL enriched with different formulations on the very same cellular system, thus reducing biological variability and limiting invasive procedures such as muscle biopsy.

Intriguingly, whileplasma bioavailability showed that both CoQ_10_ formulations were able to significantly increase plasma levels and its oxidative status in a statistically similar manner (Figure 3A,B), when we used pooled LDL isolated from the human plasma of volunteers who were treated with different formulations and then normalized for their CoQ_10_ content for the ex vivo supplementation of cultured cells, different bioavailability profiles for UBQ and CoQ_10_ formulas were observed in both cell lines used(Figure 3A,B). Specifically, UBQ-enriched LDL were more efficient in delivering CoQ_10_ in both human dermal fibroblasts and muscle myoblasts. The increase in cellular CoQ_10_ levels was significantly higher with respect to that obtained incubating cells with crystalline CoQ_10_-enriched LDL using identical CoQ_10_ dosages and times of exposure (Figure 4A). Dermal fibroblasts constitute the main cellular component of the dermis, a tissue characterized by lower CoQ_10_ content compared with other tissues, such as skeletal and cardiac muscle, and this might contribute to a higher tendency to acquire the exogenous CoQ_10_that is necessary for its critical role in cellular bioenergetics and antioxidant protection.

Surprisingly, skeletal muscle cells, which are well known for their refractoriness to exogenous CoQ_10_ uptake, also significantly increased their CoQ_10_ content when exposed to UBQ-enriched LDL at a much higher extent then the cells exposed to crystalline CoQ_10_-enriched LDL (Figure 3A,B). Moreover, UBQ in murine skeletal muscle cells was able to significantly improve cellular CoQ oxidative status, while no significant effects were observed in cells incubated with crystalline CoQ_10_-enriched LDL (Figure 5). This different redox improvement was tissue specific; in fact, in human dermal fibroblasts, both formulations were equally effective at increasing the percentage of cellular reduced CoQ_10_ oxidative status (Figure 5).

Enhanced muscle delivery of CoQ_10_ provided by Ubiqsome could be associated with the physio–chemical properties of the lipid components of phytosomes that could be better absorbed by plasma membranes of muscle cells or to more complex biochemical processes possibly linked with a promotion of mitochondrial biogenesis.

Concerning the phytosome composition, we did not analyze the protein and lipid composition of the pooled Ubiqsome-LDLs which could play a critical role. We aim to address this in future studies. Furthermore, other functional parameters related to mitochondrial/cellular oxidative statuses and oxidative stress resistance, which were not investigated in the present work, will be addressed in future experiments in order to validate the effect of increased CoQ bioavailability on cellular metabolism.

Indeed, mitochondria-rich tissues are characterized by elevated CoQ_10_ content but also by their limited ability to absorb exogenous CoQ_10_. The fact that under physiological conditions, mitochondria CoQ_10_ content is not saturating but is in the range of the Km of mitochondrial respiratory complexes [42] suggests that these tissues may have developed a selective permeability to CoQ_10_ that deserves particular attention.

In line with this observation, Kamzalov et al. report that exogenous CoQ_10_ uptake in mice homogenate is tissue specific as it is at its maximum in the liver and its minimum in skeletal muscle [36]. In fact, unlike other lipophilic endogenous and dietary molecules, very little is known about specific CoQ_10_ translocation proteins. To date, molecular mechanisms involved in tissue CoQ_10_ uptake remain not fully characterized, although recent studies using different cellular models to identify novel CoQ_10_ transporters including CoQ biosynthetic proteins have been suggested to be involved in intracellular trafficking.

In particular, in yeast cells, Cqd1 and Cqd2 proteins belonging to UbiB family (homologues to human COQ8A and B) were identified as main carriers in intracellular CoQ distribution. Specifically, Cqd1 and Cqd2 have been suggested to mediate CoQ transport from the mitochondria to the cytosolic environment and viceversa [43]. Another study conducted on yeast and bacteria cells analyzed a novel CoQ_9_ protein function involved in the inner mitochondrial membrane (IMM) permeability of CoQ precursors. In fact, thanks to biochemical, structural, and computational data, the authors show how CoQ_9_ is able to deliver both CoQ precursors and promote its translocation to mitochondrial CoQ biosynthetic enzymes (CoQ_7_) [44]. Mitochondria mass might therefore represent a targetable regulator of CoQ_10_ uptake that could be triggered using inducers of mitochondrial biogenesis. Further studies are required to verify this hypothesis and to evaluate whether the induction of mitochondria biogenesis underlies increased CoQ_10_ uptake evoked by Ubiqsome.

## 5. Conclusions

This exvivo study confirms invivo evidence of improved CoQ_10_phytosome muscle bioavailability which is important considering their well-described resistance to exogenous CoQ uptake. Further experiments are required in order to provide mechanistic insights beyond phytosome-improved bioavailability. The proposed model could provide a useful tool to investigate the mechanisms underlying tissue-dependent CoQ translocation that still remain unexplained.

## Figures and Tables

**Table 1:** CoQ_9_ and CoQ_10_ values (ng/mg protein) in mouse skeletal cells that were exposed to different treatments with pooled LDL extracted from volunteers who were supplemented with Ubiqsome (UBQ) and crystallized CoQ_10_(CoQ). ^a^ *p* < 0.0001 compared with the control; ^b^ *p* < 0.0001 compared with CoQ.

Treatment	CoQ_9_ (ng/mg Protein)	CoQ_10_ (ng/mg Protein)
Ctrl	63.5 ± 42.9	2.6 ± 0.5
UBQ	83.3 ± 38.3	30.2 ± 12.6 ^(a;b)^
CoQ	68.9 ± 30.1	7.9 ± 2.8

## Data Availability

All data presented in the study are available from the corresponding author (L.T.) upon request.
